# Lactoferrin-Functionalized Noble Metal Nanoparticles as New Antivirals for HSV-2 Infection

**DOI:** 10.3390/microorganisms10010110

**Published:** 2022-01-05

**Authors:** Malgorzata Krzyzowska, Marcin Chodkowski, Martyna Janicka, Dominika Dmowska, Emilia Tomaszewska, Katarzyna Ranoszek-Soliwoda, Katarzyna Bednarczyk, Grzegorz Celichowski, Jaroslaw Grobelny

**Affiliations:** 1 Department of Nanobiology and Biomaterials, Military Institute of Hygiene and Epidemiology, 01-163 Warsaw, Poland; marcin.chodkowski@wihe.pl (M.C.); martyna.janicka@wihe.pl (M.J.); dominikadmowska99@gmail.com (D.D.); 2 Division of Microbiology, Department of Preclinical Sciences, Institute of Veterinary Medicine, Warsaw University of Life Sciences—SGGW, Ciszewskiego 8, 02‐786 Warsaw, Poland; 3 Department of Materials Technology and Chemistry, Faculty of Chemistry, University of Lodz, Pomorska 163 St., 90-236 Lodz, Poland; emilia.tomaszewska@chemia.uni.lodz.pl (E.T.); katarzyna.soliwoda@chemia.uni.lodz.pl (K.R.-S.); katarzyna.bednarczyk@chemia.uni.lodz.pl (K.B.); grzegorz.celichowski@chemia.uni.lodz.pl (G.C.); jaroslaw.grobelny@chemia.uni.lodz.pl (J.G.)

**Keywords:** herpes simplex virus type 2, lactoferrin, genital herpes, nanoparticles, microbicide

## Abstract

(1) Background: Lactoferrin has been recognized as a potent inhibitor of human herpetic viruses, such as herpes simplex type 1 (HSV-1) and 2 (HSV-2). In this work, we tested if silver and gold nanoparticles modified with lactoferrin (LF-Ag/AuNPs) can become novel microbicides with additional adjuvant properties to treat genital herpes infection. (2) Methods: The antiviral and cytotoxic activities of LF-Ag/AuNPs were tested in human skin HaCaT and vaginal VK-2-E6/E7 keratinocytes. Viral titers and immune responses after treatment with LF-Ag/AuNPs were tested in murine vaginal HSV-2 infection. (3) Results: LF-Ag/AuNPs inhibited attachment and entry of HSV-2 in human keratinocytes much better than lactoferrin. Furthermore, pretreatment with LF-AgNPs led to protection from infection. Infected mice treated intravaginally with LF-Ag/AuNPs showed lower virus titers in the vaginal tissues and spinal cords in comparison to treatment with lactoferrin. Following treatment, vaginal tissues showed a significant increase in CD8+/granzyme B + T cells, NK cells and dendritic cells in comparison to NaCl-treated group. LF-Ag/AuNPs-treated animals also showed significantly better expression of IFN-γ, CXCL9, CXCL10, and IL-1β in the vaginal tissues. (4) Conclusions: Our findings show that LF-Ag/AuNPs could become effective novel antiviral microbicides with immune-stimulant properties to be applied upon the mucosal tissues.

## 1. Introduction

Herpes simplex virus type 2 (HSV-2) is the primary cause of genital herpes and one of the most common sexually transmitted infections. After establishing latency in the sacral ganglia, HSV-2 reactivates, leading to intermittent shedding of virus in the genital tract [1]. Global prevalence of HSV-2 is high: as the majority of HSV-2 cases are subclinical and asymptomatic, viral shedding results in high rates of viral transmission. In 2012, an estimated 417 million people aged 15–49 (11%) were living with HSV-2 infection, of whom ~50% were women [2]. Epidemiological and clinical studies showed that genital herpes is positively linked to the risk of acquiring and transmitting HIV [3]. Antiviral drugs, which target viral DNA polymerase, can partially control the signs and symptoms of genital herpes when used to treat first clinical and recurrent episodes or when used as daily suppressive therapy. However, these drugs neither eradicate latent virus nor affect the risk, frequency, or severity of recurrences after the drug is discontinued [1]. 

Several natural antimicrobial proteins and peptides have demonstrated the ability to inhibit viral infection and block viral entry into the host cell or influence later stages of viral growth [4]. Lactoferrin (LF) is a glycoprotein present in external secretions, having multiple functions, including antiviral activity and stimulation of immune response [5,6]. LF has shown antiviral activity against several viruses, including rotavirus, respiratory syncytial virus, hepatitis C virus (HCV), herpes virus, and HIV [4,7,8,9]. LF exerts its anti-HSV effect by inhibiting viral attachment and entry into the host cell [4,10,11]. Antiviral activity of LF has been showed against both HSV-1 and HSV-2, although the exact antiviral mechanism is slightly different, due to the difference between the two viruses in the initial binding with target cells [1,4,6]. 

Silver nanoparticles (AgNPs) have been demonstrated to exert an antiviral activity against several viruses [12]. There are two ways the AgNP interacts with the pathogenic virus: (1) the AgNP will bind to the outer coat of the virus thus inhibit the attachment of virus towards cell receptors and (2) the AgNP will bind to the DNA or the RNA of the virus thus inhibiting the replication or propagation of the virus inside the host cells [12,13]. 

We have previously described anti-HSV-2 activity of tannic acid modified AgNPs (TA-AgNPs) by using both in vitro and in vivo models [14]. The mechanisms of antiviral action of TA-AgNPs included blocking of virus attachment, entry, cell-to-cell spread as well as induction of anti-viral cytokine and chemokine production [1]. Moreover, anti-viral effect was size-dependent, with smaller nanoparticles showing stronger activation of inflammatory reaction [14].

Recently, LF has been attached onto the surface of biosynthesized AgNPs showing higher antibacterial activity against selected bacterial strains as than their single components [15]. In this study, we tested whether combined activities of lactoferrin and silver or gold nanoparticles of different sizes can lead to development of a novel microbicide using both in vitro and in vivo model of HSV-2 infection. 

## 2. Materials and Methods

### 2.1. Synthesis of Nanoparticles

The nanoparticles colloids were synthesized in water according to chemical reduction method. The following reagents for the nanoparticles and nanoconjugates preparation were used: tetrachloroauric acid (HAuCl_4_, Sigma-Aldrich, ≥49%), tannic acid (C_76_H_52_O_46_, Sigma-Aldrich), sodium citrate (C_6_H_5_Na_3_O_7_·2H_2_O) purity 99.0%, Sigma-Aldrich), silver nitrate (AgNO_3_, purity 99.999%, Sigma-Aldrich, St. Louis, MO, USA), sodium borohydride (NaBH_4_, purity ≥ 96%, Sigma-Aldrich), lactoferrin (CAS no. 936541-36-5. Sigma Aldrich ≥ 85%). For all preparations, deionized water obtained from Deionizer Millipore Simplicity UV system (specific resistivity of water was 18.2 MΩ·cm) was used. 10 nm AgNPs were prepared by reduction of silver nitrate with sodium borohydride in the presence of sodium citrate and tannic acid. The molar ratio of silver nitrate: sodium citrate: tannic acid: sodium borohydride reagents was equal to 1:7:2:4. For the synthesis process, silver nitrate (1.574 g, 1%) and deionized water (92.914 g) were mixed in the flask for 5 min, then a mixture of sodium citrate (4.181 g, 4%) and tannic acid (0.630 g, 5%) was added. After ~2s, sodium borohydride (0.701 g, 2%) was added and the solution was mixed for additional 15 min. The final concentration of metal NPs was equal 100 ppm.

10 nm AuNPs were prepared by reduction of tetrachloroauric acid with a mixture of sodium citrate and tannic acid with the molar ratio of the reagents equal 1:3:2. Briefly, tetrachloroauric acid (12.691 g, 0.136%) and deionized water (81.655 g) were warmed to the boiling point under reflux and then a mixture of reducing and stabilizing agents was added (mixture of sodium citrate 3.929 g, 1% and tannic acid 1.725 g, 1%). The mixture was boiled for additional 15 min and then cooled down to room temperature. The final concentration of metal NPs was equal 100 ppm. 30 nm AgNPs were prepared by reduction of silver nitrate with a mixture of sodium citrate and tannic acid (the molar ratio of reagents was equal 1:7:2). The synthesis procedure was as follows: silver nitrate (1.574 g, 1%) and deionized water (93.615 g) were mixed and boiled under reflux. Next, a warm mixture of sodium citrate (4.181 g, 4%) and tannic acid (0.630 g, 5%) was added to the boiling solution and heated for an additional 15 min, and then the colloid was cooled down to room temperature. The final concentration of metal NPs was equal 100 ppm. Nanoconjugates were prepared by incubation of nanoparticles with lactoferrin (1% aqueous solution, human recombinant, iron-saturated, Sigma-Aldrich) at room temperature for 24 h. Briefly, 2.475 g of each colloid was incubated with 27.76 mg of 1% aqueous solution of lactoferrin to obtain the final lactoferrin concentration equal 110 µg/mL for each sample. Attempts to prepare 30 nm AuNPs conjugated with lactoferrin failed due to instability of the obtained colloid. For all conjugates a reference sample was prepared as an aqueous solution of lactoferrin in concentration corresponding to concentration present in colloids. The nanoparticles after modification with lactoferrin (LF) were denoted as LF-Ag/AuNPs, while unmodified as Ag/AuNPs.

Nanoparticles before and after lactoferrin functionalization were characterized using the following techniques: high-resolution scanning electron microscopy (HR-SEM equipped with STEM II detector; NovaNanoSEM 450, FEI, Hillsboro, Oregon, USA; images acquired at bright field (BF), dark field (DF), high-angle annular dark-field (HAADF) detector mods; samples for measurements prepared by drop-casting of colloid on carbon coated copper grids), DLS and Zeta (Lifesizer 500, Particle Analyzer, Anton Paar, Graz, Austria), UV–Vis (spectrophotometer UV-5600, Biosens, METASH, Shanghai, China).

### 2.2. Virus and Anti-Viral Tests

HSV-2 (strain 333) was grown and titrated in Vero cells (ATCC^®^ CCL-81, Rockville, MD, USA) and kept at −80 °C until use. Virus was diluted in MEM (Thermo Fisher Scientific, Waltham, MA, USA) and maintained on ice until administered to mice or mixed with nanoparticles maximum one hour later. To analyze how **LF-**NPs can influence viral attachment, cells were pre-chilled at 4 °C for 15 min, then co-treated with NPs and HSV-2 for 1 h. After this time, virus and NPs were removed and cell monolayers were washed with ice-cold PBS, and further incubated at 37 °C. At 24 h post infection, virus titers were determined by plaque assays or qPCR (see below), as described previously [14]. The viral penetration assay started by pre-chilling cells at 4 °C for 15 min and then cells were infected for 2 h at 4 °C to allow virus binding but not entry. The inoculum was removed, and cells were washed with ice-cold PBS before adding **LF-**NPs for 2 h at 37 °C. The **LF-**NPs were afterwards removed, and cells were washed twice with cold PBS. After another 18 h at 37 °C, virus titers were determined by qPCR. For examining post viral entry effects of **LF-**NPs use, cells were infected at 37 °C, then the virus was removed, cells washed, and **LF-**NPs were added 6 h post infection (p.i.). At 24 h post infection, the infected cultures were analyzed by qPCR. Pre-treatment was performed by incubating cells with **LF-**NPs (at 37 °C) for 6 h then cells were washed, infected and further titered by qPCR.

### 2.3. Cell Lines and Infection

Human VK2-E6/E7 vaginal epithelial cells were obtained from ATCC (CRL-2616) and propagated in EpiLife™ medium supplemented with EpiLife™ Defined Growth Supplement (EDGS) (Thermo Fisher Scientific) and 10 U/mL penicillin and 100 μg/mL streptomycin (GIBCO by Thermo Fisher Scientific). Human HaCaT keratinocytes were from CLS Cell Lines Service GmbH (Eppelheim, Germany) and propagated in Dulbecco’s modified EMEM (DMEM) supplemented with 10% fetal calf serum, 10 U/mL penicillin, and 100 μg/mL streptomycin (GIBCO). Both cell lines were maintained in standard conditions (37 °C, 5% CO_2_) and handled according to ATCC and CLS recommendations. 

### 2.4. Cell Viability Assay

Cell viability was measured using the MTT assay ((-(4, 5-Dimethyl-2-thiazolyl)-2, 5-diphenyl-2H-tetrazolium bromide, Sigma-Aldrich). Cultures of Vero, HaCaT and VK-2-E6/E7 A were incubated for 24 h at 37 °C in a 96-well plate with lactoferrin modified NPs and lactoferrin in a range of NPs concentrations (1–50 μg/mL) in a final volume of 100 µL. The control cells were untreated. Thereafter, 20 µL MTT at a concentration of 5 mg/mL in PBS was added to each well, and the cells were incubated for 4 h at 37 °C. Subsequently, 100 µL of 10% Triton X-100, 0.1 M HCl in isopropanol solution was added, and the cells were incubated for 30 min at room temperature. After the incubation, the optical density (OD) was measured at a wavelength of 570 nm using Omega Microplate Spectrophotometer (BioTek Instruments, Inc., Winooski, VT, USA). The cellular viability was calculated as the percentage of viable cells relative to the control. All experiments were performed in triplicate. The 50% cytotoxic concentration (CC_50_) was defined as the compound’s concentration (μg/mL) required for the reduction of cell viability by 50%, which were calculated by regression analysis, plotting cytotoxicity percentage to NPs concentration. MNTC (maximum non-toxic concentrations) was calculated as NPs concentration with ≤20% of non-viable cells and these concentrations were used for in vitro studies.

### 2.5. Virus Titration

Total DNA and RNA from vaginal tissues and spinal cords were isolated at day 7 post HSV-2 infection using RNA/DNA Extracol kit (Eurx, Gdansk, Poland). DNA from infected cell cultures was isolated with Universal RNA/DNA extraction kit (Eurx). HSV-2 was detected by qPCR with primers and probe for the viral envelope glycoprotein (gB), as described by Namvar et al. [16] in Rotor-Gene Q (Qiagen, Hilden, Germany) with GoTaq^®^ Probe qPCR Master Mix (Promega, Madison, WI, USA). Forward primer: ‘TGCAGTTTACGTATAACCACATACAGC’, reverse primer: ‘AGCTTGCGGGCCTCGTT’ and probe: 5′ FAM-CGCCCCAGCATGTCGTTCACGT’. Standard curve analysis was based on Ct values and serial of 10-fold dilutions of the plasmid standard containing the gB gene with an initial concentration of 2.62 × 10^6^ HSV-2 genome copy numbers per reaction. A standard curve was included in each PCR run. Data are expressed as the HSV-2 copy number per ng of the total DNA in the tissue. 

### 2.6. Ethical Statement

The study was performed in accordance with the recommendations of the Polish Act of 21 January 2005 on animal experiments (OJ no 33, item 289) and Directive 2010/63/EU of the European Parliament and the Council of 22 September 2010 on the protection of animals used for scientific purposes. The protocol was approved by the Local Committee on the Ethics of Animal Experiments in Warsaw, Poland (permit Number: WAW2/69/2021).

### 2.7. Genital HSV-2 Infection and Virus Challenge

Female C57BL/6 mice, 6 to 8 weeks old, were injected s.c. with 2.0 mg/mouse of medroxyprogesterone (Depo-Provera; Pfizer, Puurs, Belgium) in 200 µL of PBS. Five days later the mice were anesthetized and inoculated intravaginally with 20 μL of virus solution, containing 10^3^ PFU of HSV-2 strain 333 in MEM medium [14,17]. Vaginal washings were performed at 24, 48 and 72 h after infection with 100 μL of 0.9% NaCl solution containing 10 μg/mL LF-Ag/AuNPs or 0.9% NaCl solution. Illness was scored according to the scale: no signs = 0; slight inflammation of anogenital area = 1; gross inflammation = 2; gross inflammation and hair loss = 3; and gross inflammation, ulceration and neurological signs = 4. Animal scores were averaged in each group to obtain a single representative value. At day 7 post infection, mice were sacrificed by cervical dislocation and the spinal cords and vaginas were collected for further tests. 

### 2.8. Flow Cytometry Phenotypic Analysis

Cell suspensions from vaginal tissues were prepared as follows: tissues were cut into small pieces and treated with Liberase TL (Roche, Indianapolis, IN, USA) in MEM medium at 37 °C for 40 min. Tissues were next pressed through a 70 µm cell strainer and washed in PBS/1%FBS. Cell suspensions were pretreated with the Fc receptors block-rat anti-CD16/32 antibody (2.4G2) (BD Biosciences, Franklin Lakes, NJ, USA) according to the manufacturer’s protocol. The following antibodies were used: anti-CD3-FITC (145-2C11, ThermoFisher Scientific), anti-CD4-PE (clone RM4-5, BD Biosciences), anti-CD8-PerCP (clone 53-6.7., BD Biosciences), anti-NK1.1-APC (clone PK136, BD Biosciences), anti-CD11b-FITC or PE (clone M1/70) (BD Biosciences), anti-CD69-APC antibody (H1.2F3, eBioscience), anti-CD11c-APC (HL3, eBioscience), rat anti-F4/80-FITC (BM8, eBioscience) and rat anti-I-A-PE (M5/114.15.2, eBioscience) antibodies, and rat anti-Gr-1-PE (RB6-8C5, BD Biosciences). Following the immunolabeling for the extracellular markers, cells were fixed with Perm/Wash buffer (BD Bioscience) and were incubated with anti-IFN-γ APC (eBioscience, clone-XMG1.2). The stained cell suspensions were analyzed in FACS Calibur for the percentage of positively stained cells and analyzed using FlowJo software (Tree Star, Ashland, OR, USA).

### 2.9. qPCR for Cytokines and Chemokines

Approximately 0.5 μg of total RNA isolated from vaginal tissues and spinal cords, as described above, was converted to cDNA using MLV Reverse transcriptase (Invitrogen, Thermofisher Scientific). qPCR reactions for cytokines and chemokines were carried out using GoTaq^®^ Probe qPCR Master Mix (Promega) and TaqMan^®^ probes for the detection of IL-1β (Mm00434228_m1), IFN-γ (Mm01168134_m1), TNF-α (Mm00443258_m1), CXCL9 (Mm00434946_m1), CXCL10 (Mm00445235_m1), and GADPH (Mm99999915_g1) in Rotor-Gene Q (Qiagen). Cytokines and chemokines were normalized to the mean threshold cycle (CT) of GADPH housekeeping gene. The 2^−∆∆Ct^ method was used in calculating the relative ratio to control uninfected tissue. 

### 2.10. Statistical Methods

For statistical analysis, GraphPad Prism version 7 (GraphPad software) was used. Data were analyzed using an unpaired Student’s t-test (normal distribution) or the Mann–Whitney U test and the results are reported as mean ± standard error of the mean (SEM) unless indicated otherwise. The *p* < 0.05 was considered statistically significant.

## 3. Results

### 3.1. Characterization of Nanoparticles

**Figure 1:**
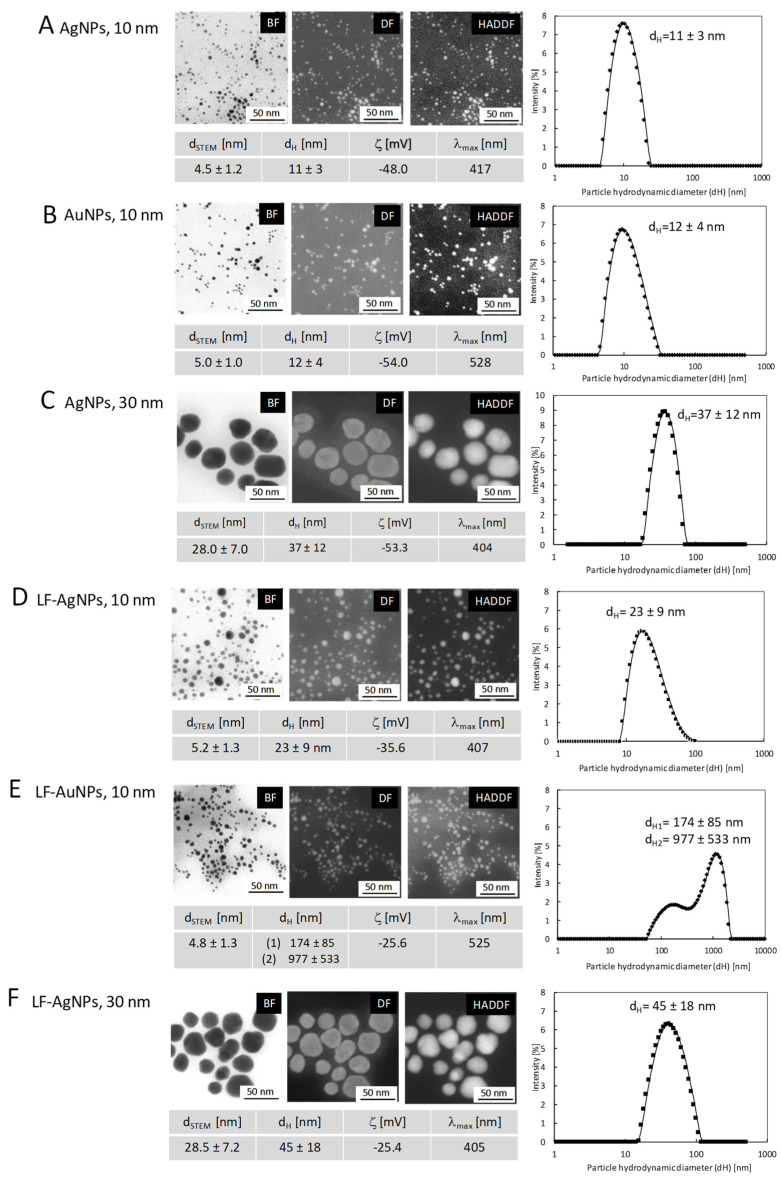
HR-SEM image, DLS size distribution histogram, and overall results of nanoparticles and nanoconjugates characterization results of NPs (**A**–**C**) and LF-NPs conjugates (**D**–**F**) (d_STEM_—the mean size of metallic core; d_H_—hydrodynamic particles size; ζ—Zeta potential; λ_max_ absorption band maximum).

Microscopic and spectroscopic measurements were performed to characterize size, shape, size distribution and colloidal stability of nanoparticles as well as to determine and confirm the stability of nanoconjugates after lactoferrin functionalization (Figure 1). UV–Vis spectroscopy was used for confirmation of NPs synthesis by observation of the characteristic localized surface plasmon resonance (LSPR) peak in UV–Vis spectra. For all colloids, the required peak was observed in the region characteristic for specific nanoparticles: for AgNPs 10 nm at λ_max_ = 417 nm, for AuNPs 10 nm at λ_max_ = 528, for AgNPs 30 nm at λ_max_ = 404 nm. The hydrodynamic size of nanoparticles and its colloidal stability were tested with DLS and Zeta potential measurements. The DLS results confirmed stability of nanoparticles with the hydrodynamic particle size equal: d_H1_ = 11 ± 3 nm; d_H2_ = 12 ± 4 nm; d_H3_ = 37 ± 12 nm and Zeta potential equal: ζ_1_ = −48.0 mV, ζ_2_ = −54.0 mV and ζ_3_ = −53.3 mV; for colloids AgNPs 10 nm (Figure 1A), AuNPs 10 nm (Figure 1B) and AgNPs 30 nm (Figure 1C), respectively. HR-SEM measurement revealed the spherical shape of all nanoparticles with the mean size of metallic core equal: d_1STEM_ = 4.5 ± 1.2 nm, d_2STEM_ = 5 ± 1 nm, d_3STEM_ = 28 ± 7 nm for colloids, which for consistency are called AgNPs 10 nm, AuNPs 10 nm and AgNPs 30 nm, respectively. The size distribution of the NPs was analyzed by measurement of at least 300 nanoparticles for each sample and the histogram for the size distribution of particles was prepared.

The colloidal stability of nanoparticles after functionalization with lactoferrin (LF-NPs) was monitored with UV–Vis, HR-STEM, DLS, and Zeta potential measurements. UV–Vis spectroscopy revealed the presence of a peak in the region characteristic for spherical silver nanoparticles at λ_max_ = 407 nm and λ_max_ = 405 nm for LF-AgNPs 10 nm and LF-AgNPs 30 nm conjugates, respectively. For LF-AuNPs 10 nm, the λ_max_ was observed in the region typical for spherical gold nanoparticles at 525 nm. Those values only slightly differ from the values of λ_max_ obtained for the aqueous colloids, which is related with the change in the environment of NPs due to functionalization with lactoferrin. DLS measurements revealed an increase in the hydrodynamic diameter of nanoparticles after functionalization from d_H1_ = 11 ± 3 nm to d_H1-LF_ = 23 ± 9 nm; from d_H2_ = 12 ± 4 nm to d_H2-LF_ = 177 ± 85 nm and 977 ± 533 nm; from d_H3_ = 37 ± 12 nm to d_H-LF3_ = 45 ± 18 nm for LF-AgNPs 10 nm (Figure 1D), LF-AuNPs 10 nm (Figure 1E) and LF-AgNPs 30 nm (Figure 1F) conjugates, respectively. The d_H_ peaks observed for LF-AuNPs 10 nm conjugates at values above 100 nm (at 177 nm and 977 nm) are related to the presence of nanoparticle conglomerates in the colloid. 

It is possible that the conglomerates of nanoparticles are present in the colloid, moving as a “cloud” of single nanoparticles, and during the measurement of the hydrodynamic particle diameter they give a signal that is detected as a large object. However, the HR-SEM images (Figure 1D–F) clearly show that all nanoparticles after lactoferrin functionalization retain stability and no agglomeration processes are observed, also in the case of sample LF-AuNPs 10 nm (Figure 1E HR-SEM images). Thus, those results confirm our notion that the DLS signal obtained for the LF-AuNPs 10 nm comes from the conglomerates of individual particles formed in the solution (HR-SEM images confirm no metallic connections between individual nanoparticles in conglomerates). 

The mean size of metallic core after functionalization remains unchanged for all conjugates. The Zeta potential for conjugates is equal: ζ_1_-_LF_ = −35.6 mV, ζ_1_-_LF_ = −25.6 mV, and ζ_1_-_LF_ = −25.4 mV, for LF-AgNPs 10 nm, LF-AuNPs 10 nm, and LF-AgNPs 30 nm, respectively. In summary, the UV–Vis, DLS, Zeta potential, and HR-SEM results of the nanoparticles and nanoconjugates confirm the colloidal stability of nanoparticles before and after lactoferrin functionalization.

### 3.2. Antiviral Effects of Lactoferrin Conjugates with Nanoparticles In Vitro

To ensure that tested Ag/AuNPs concentrations were non-toxic, cytotoxicity of nanoparticles in Vero, VK-2 and HaCaT cell lines was assessed using MTT assay (Table 1). The results of the MTT assay showed that all tested LF-modified NPs were slightly more toxic to VK2-E6/E7 than to Vero and HaCaT cells (Table 1) and their toxicity did not differ from unmodified Ag/AuNPs. The 50% cytotoxic concentrations (CC_50_) for 10 nm AgNPs were approx. 2 times lower than those for 10 nm AuNPLactoferrin-Modified Ag/AuNPs Reduce HSV-2 Infection In Vivos and 30 nm AgNPs (Table 1). However, the MNTC (maximum non-toxic concentrations) showed values lower than CC_50_ (Table 1). Therefore, for in vitro infectious assays we chose concentration of 7.5 µg/mL for LF-modified or unmodified 10 nm AuNPs and 30 nm AgNPs, and 5 µg/mL for LF-modified or unmodified 10 nm AgNPs. 

**Figure 2:**
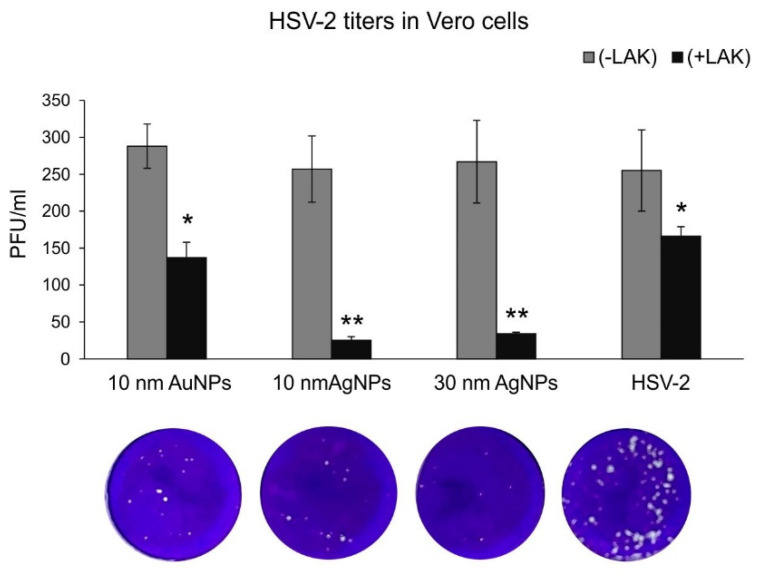
Inactivation of HSV-2 infection by lactoferrin-modified AgNPs or AuNPs in Vero cells is metal-related. HSV-2 titers (PFU/mL) in Vero cells infected with HSV-2 pre-incubated for 1 h with 10 nm AuNPs or AgNPs and 30 nm AgNPs modified or unmodified with lactoferrin. The data are expressed as means from three independent experiments ± SEM. * represents significant differences with *p* ≤ 0.05, ** *p* ≤ 0.001 in comparison to infected control (Student *t* test). Lower panel shows photos of crystal violet stained wells corresponding to graphs.

The antiviral properties of LF-modified Ag/AuNPs in Vero cell culture were tested by standard plaque forming assay (PFU/mL). Incubation of HSV-2 with different LF-modified Ag/AuNPs for 60 min prior to infection showed a size-dependent virus inactivation by AgNPs sized 10 and 30 nm (*p* ≤ 0.001) (Figure 2) and much less efficient virus inhibition by LF modified AuNPs sized 10 nm (*p* = 0.042) (Figure 2). Of all tested AgNPs sizes, 30 nm showed the highest antiviral activity in comparison to HSV-2 infected cultures (48 ± 5.2 and 255.4 ± 24.42 PFU/mL, respectively) (Figure 2). The same antiviral tests in Vero cells were performed using unmodified Ag/AuNPs (Figure 2) and showed no significant inhibition of HSV-2 infection. Furthermore, LF solution with concentration corresponding to the concentration of LF used to modify showed significant inhibition of HSV-2 infection, albeit not as efficient as LF-AgNPs (*p* = 0.039) (Figure 2).

### 3.3. Lactoferrin-Modified Ag/AuNPs Show Differentiated Antiviral Mechanisms Depending on the Keratinocyte Origin

**Figure 3:**
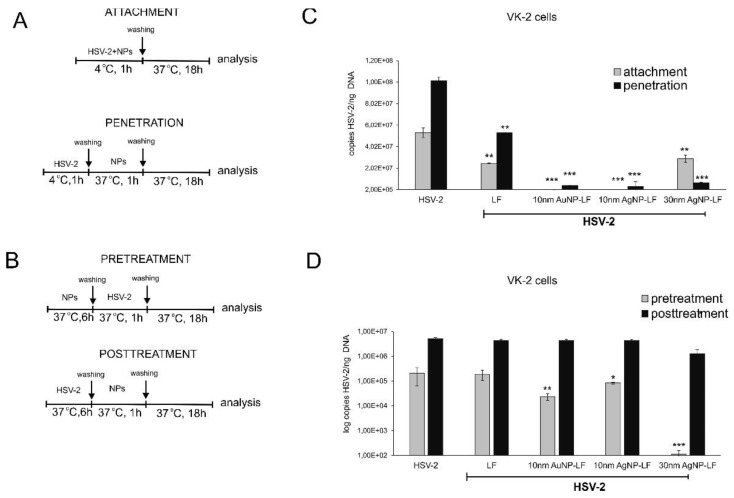
Lactoferrin-modified Ag/AuNPs block HSV-2 attachment and penetration in vaginal VK-2-E6/E7 keratinocytes. (**A**,**B**) Schematic representation of test procedures, I inhibition of virus attachment and penetration in VK-2-E6/E7 cell cultures with the use of 10 nm LF-Ag/AuNPs, 30 nm LF-AgNPs or lactoferrin. At 24 h p.i. cells were subjected to HSV-2 copies titration by qPCR. (**C**,**D**) efficiency of pre- or post-treatment with 10 nm LF-Ag/AuNPs, 30 nm LF-AgNPs or lactoferrin was tested 6 h pre- or post-infection. At 24 h p.i. cells were subjected to HSV-2 copies titration by qPCR. In all experiments, lactoferrin in concentrations corresponding to the amount used to modify NPs served as positive control. The data are expressed as means from three independent experiments ± SEM. * represents significant differences with *p* ≤ 0.05, ** *p* ≤ 0.01, *** *p* ≤ 0.001 in comparison to untreated infected control (Student *t* test).

To understand the antiviral mechanism(s) involved, we tested the effect of lactoferrin modified Ag/AuNPs against HSV-2 attachment to the host cell surface and subsequent membrane fusion in two types of human keratinocytes—skin-derived HaCaT cells and vaginal VK-2-E7/E7 cells. The details of the procedure are described in Figure 3. All lactoferrin-modified Ag/AuNPs prevented attachment of HSV-2 to VK-2-E6/E7 cells, albeit LF-modified 30 nm AgNPs showed the same efficiency as lactoferrin itself (Figure 3A). The most effective were 10 nm LF-modified Au/AgNPs (*p* ≤ 0.001) (Figure 3A). To test if LF-modified Ag/AuNPs can impair the penetration phase, HSV-2 was allowed to bind to the cell surface at 4 °C and then to penetrate keratinocytes by a temperature shift to 37 °C in the presence or absence of the tested Ag/AuNPs or lactoferrin (Figure 3A). All tested LF-modified Ag/AuNPs impaired virus entry into VK-2 cells with ~90% efficiency, although no size/metal-related differences were observed (*p* ≤ 0.001) (Figure 3A). Lactoferrin also blocked viral penetration in a significant manner (*p* = 0.009) (Figure 3A). 

To investigate the possibility of blocking cellular receptors with LF-modified Ag/AuNPs, we preincubated VK-2 cells prior to infection with NPs or lactoferrin for 6 h. Preincubation of the cells with LF-modified 30 nm AgNPs led to significant inhibition of HSV-2 infection by 75% in comparison to untreated or LF-treated infected control (*p* ≤ 0.001) (Figure 3B). Furthermore, 10 nm LF-modified Ag/AuNPs decreased infection, however, the effect was smaller (*p* ≤ 0.05) (Figure 3B). Post-treatment of VK-2-E7/E7 cells with LF-Ag/AuNPs at 6 h post infection (p.i.) showed that only 30 nm LF-AgNPs can significantly decrease virus titers (*p* ≤ 0.05) (Figure 3B).

**Figure 4:**
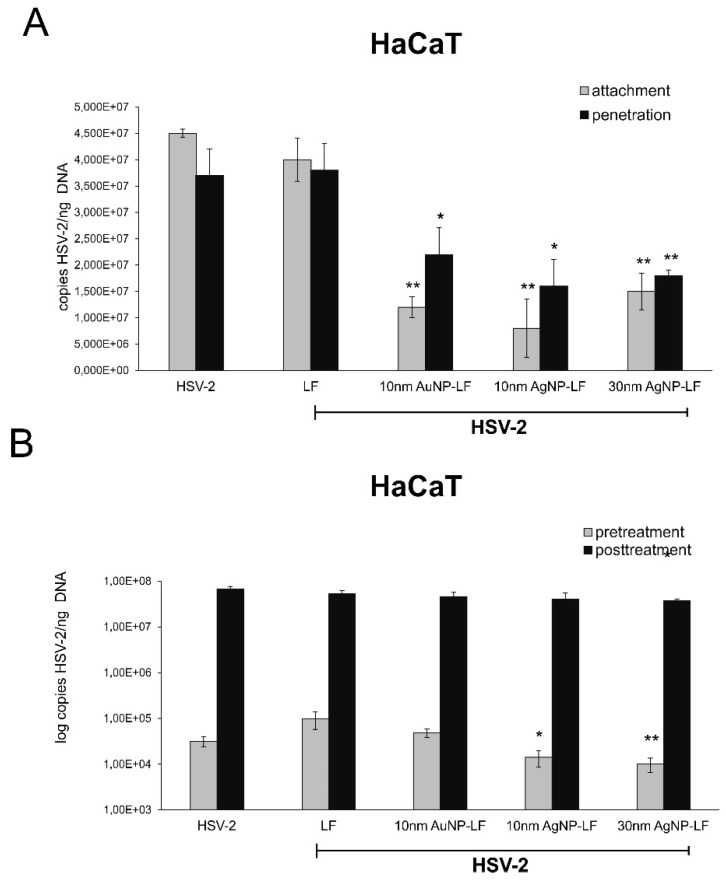
Lactoferrin-modified Ag/AuNPs block HSV-2 attachment and penetration in skin HaCaT keratinocytes. (**A**) Inhibition of virus attachment and penetration in HaCaT cell cultures with the use of 10 nm LF-Ag/AuNPs, 30 nm LF-AgNPs or lactoferrin. At 24 h p.i. cells were subjected to HSV-2 copies titration by qPCR. (**B**) efficiency of pre- or posttreatment with 10 nm LF-Ag/AuNPs, 30 nm LF-AgNPs or lactoferrin was tested 6 h pre- or post-infection. At 24 h p.i. cells were subjected to HSV-2 copies titration by qPCR. In all experiments, lactoferrin in concentration corresponding to the amount used to modify NPs served as positive control. The data are expressed as means from three independent experiments ± SEM. * represents significant differences with *p* ≤ 0.05, ** *p* ≤ 0.01 in comparison to untreated infected control (Student *t* test).

As HSV-2 also causes blisters within the anogenital area, we tested the effect of lactoferrin modified Ag/AuNPs against HSV-2 infection of skin HaCaT keratinocytes (Figure 4). All experiments were performed in the same manner as in Figure 3. In contrast to VK-2-E7/E7 cells, lactoferrin did not inhibit HSV-2 attachment or inhibition (Figure 4A) or had any antiviral effect when used in pretreatment or post-treatment (Figure 4B). All lactoferrin-modified Ag/AuNPs significantly prevented both attachment and penetration of HSV-2 to HaCaT cells (*p* ≤ 0.05) (Figure 4A). LF-modified 10 and 30 nm AgNPs showed significant inhibitory effect upon HSV-2 infection when used for pretreatment (*p* ≤ 0.05) (Figure 4A). No antiviral effect was observed when NPs were used to treat infected HaCaT cells at 6 h p.i. (Figure 4B). 

### 3.4. Lactoferrin-Modified Ag/AuNPs Reduce HSV-2 Infection In Vivo

**Figure 5:**
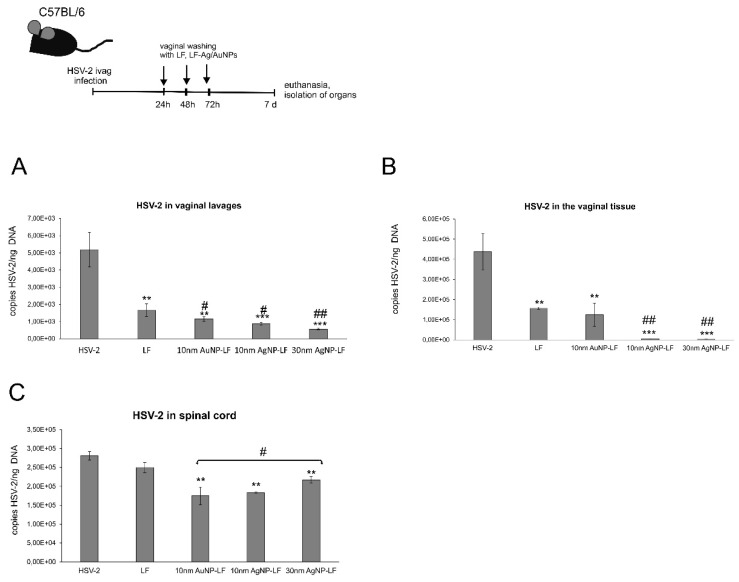
Treatment with lactoferrin-modified Ag/AuNPs reduce HSV-2 infection in vivo. C57BL/6 mice infected with HSV-2 were treated three times every 24h with 10 nm LF-Ag/AuNPs, 30 nm LF-AgNPs or lactoferrin (1 μg/mouse). (**A**) Vaginal lavages collected at 72 h p.i. (**B**) Vaginal tissues and (**C**) spinal cords isolated at 7 d p.i. were subjected to measurement of HSV-2 gB titers (copies/ng DNA) by qPCR (*N* = 10). The bars represent means ± SEM. * represents significant differences with ** *p* ≤ 0.01 and *** *p* ≤ 0.001 in comparison to untreated HSV-2 tissues. ^#^ represents significant differences with *p* ≤ 0.05, ^##^ *p* ≤ 0.01 in comparison to lactoferrin treated HSV-2 infected tissues (Mann–Whitney U test).

To confirm our in vitro findings, we employed the well-established murine model of HSV-2 genital infection [17,18]. We used LF-Ag/AuNPs as an antiviral agent in animals with on-going intravaginal primary HSV-2 infection. To study the effects of post-infection treatment, animals were infected with untreated HSV-2 and then the vaginal tissues were irrigated three times with 10 μg/mL of Lactoferrin, 10 nm LF-AuNPs, 10 nm LF-AgNPs, and 30 nm LF-AgNPs (Figure 5). We measured HSV-2 titers with qPCR in the vaginal tissues and spinal cords at 7 d p.i. and in the vaginal lavages collected at 72 h p.i. (Figure 5).

Post-infection treatment with lactoferrin resulted in a significant decrease of the viral titers in vaginal lavages and vaginal tissues (*p* ≤ 0.01) (Figure 5A,B) but not in the spinal cords (Figure 5C). The virus titers were significantly reduced for treatment with all NPs in vaginal lavages and spinal cords in comparison to untreated HSV-2 infected control and lactoferrin treatment (*p* ≤ 0.05) (Figure 5A,C). All lactoferrin conjugates significantly reduced viral titers in all tested sample types (*p* ≤ 0.05) (Figure 5A–C). Both sizes of silver nanoparticles modified with LF showed the most efficient reduction of HSV-2 titers in the vaginal tissues and vaginal lavages (*p* ≤ 0.001) (Figure 5B). 

### 3.5. Lactoferrin-Modified Ag/AuNPs Help to Activate Early Antiviral Response

**Figure 6:**
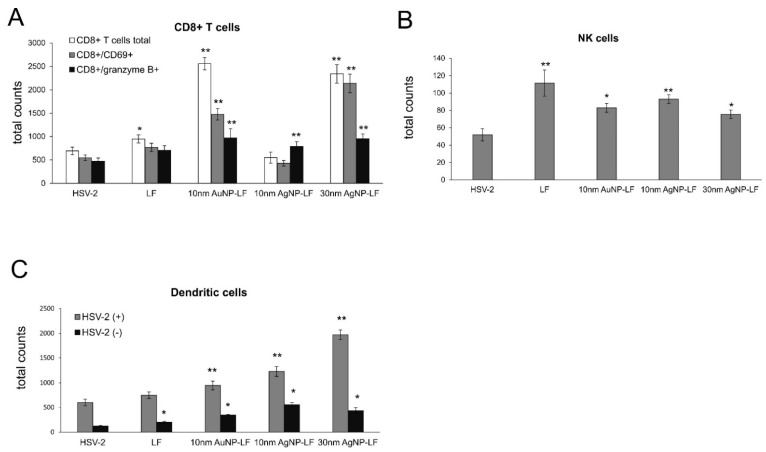
Lactoferrin-modified Ag/AuNPs activate early antiviral response in HSV-2 vaginal infection. Total counts of (**A**) CD8+ T cells, CD8+CD69+ T cells, CD8+/granzymeB+ T cells, (**B**) NK cells, and (**C**) dendritic cells in the vaginal tissue treated with NaCl or 10 nm LF-Ag/AuNPs, 30 nm LF-AgNPs or lactoferrin at 24, 48 and 72 h p.i. and isolated for further assays at 7 day p.i. Results are expressed as mean +/− SEM for *N* = 7. * represents significant differences with *p* ≤ 0.05, ** *p* ≤ 0.01 in comparison to untreated HSV-2 tissues (Student *t* test).

Here, we checked if lactoferrin-modified Ag/AuNPs can contribute to activation of early antiviral immune response. Cell suspensions prepared from vaginal tissues were subjected to immunophenotyping for CD4+ T cells, CD8+ T cells, NK cells, CD8+CD69+ T cells, and dendritic cells (Figure 6). Treatment with lactoferrin-modified 10 nm AuNPs and 30 nm AgNPs and lactoferrin early after infection led to significant increase in the total counts of CD8 T+ cells (*p* ≤ 0.05) (Figure 6A), followed by significantly increased total counts of CD8+/CD69+ T cells by lactoferrin-modified 10 nm AuNPs and 30 nm AgNPs (*p* ≤ 0.05) (Figure 6A). Interestingly, vaginal tissues treated with all NPs showed significant increase of cytotoxic CD8+/granzyme B+ T cells (*p* ≤ 0.05) (Figure 6A). No differences in CD4+ T cells counts were detected (data not shown). Furthermore, both lactoferrin and lactoferrin modified Ag/AuNPs significantly increased the total numbers of NK cells in the vaginal tissues at 7 d p.i. (*p* ≤ 0.05) (Figure 6B). The uninfected vaginal tissues treated with LF-Ag/AuNPs showed no increases in the counts of all types of T cells and NK cells (data not shown). Interestingly, both lactoferrin and LF-Au/AgNPs induced significant increase in the counts of dendritic cells in the uninfected vaginal tissues (*p* ≤ 0.05) (Figure 6C), while only LF-modified nanoparticles significantly increased numbers of dendritic cells in the HSV-2-infected vaginal tissues (*p* ≤ 0.01) (Figure 6C).

**Figure 7:**
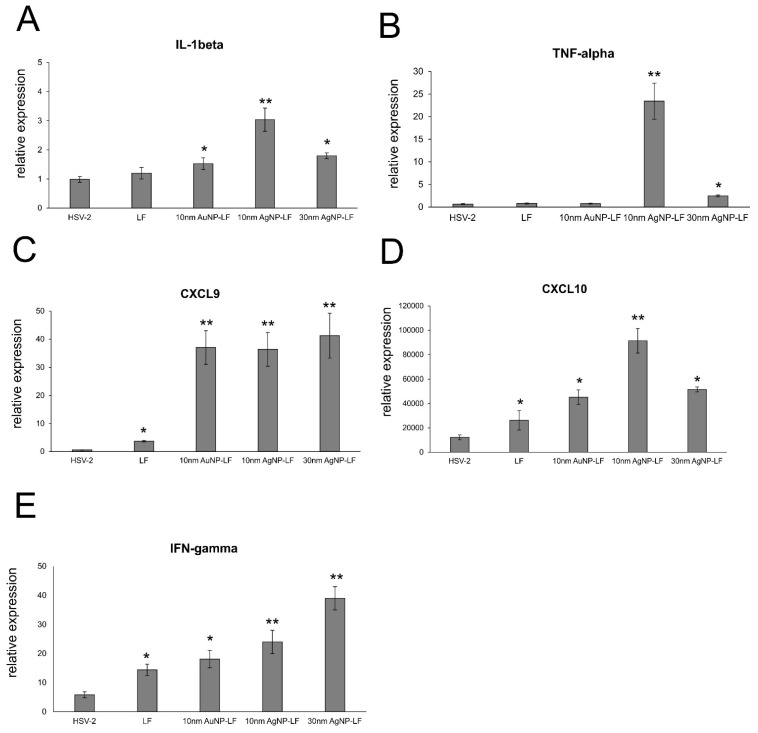
Cytokine and chemokine expression changes in the vaginal tissues at 7 day p.i. and treatment with lactoferrin modified Ag/AuNPs. Levels of IL-1β (**A**), TNF-α (**B**), CXCL9 (**C**), CXCL10 (**D**), and IFN-γ (**E**) mRNA are shown as expression relative to control on the basis of the 2^∆∆Ct^ method. N = 7. * represents significant differences with *p* ≤ 0.05, ** *p* ≤ 0.01 in comparison to untreated HSV-2 tissues.

Furthermore, we measured levels of cytokines and chemokines important in raising anti-HSV-2 antiviral response (IFN-γ, CXCL9, CXCL10) and inflammatory response (IL-1β, TNF-α) (Figure 7) in the vaginal tissues at 7 d p.i. Upon treatment with LF-modified Ag/AuNPs in HSV-2 infection, the vaginal tissue upregulated expression of IL-β mRNA with the highest upregulation observed for 10 nm LF-AgNPs (*p* ≤ 0.05) (Figure 7A). Small LF-AgNPs also significantly upregulated expression of TNF-α mRNA in the vaginal tissues (*p* = 0.01), as well as 30 nm LF-AgNPs, albeit to a lesser degree (*p* = 0.049) (Figure 7B). Both lactoferrin and lactoferrin modified Ag/AuNPs significantly upregulated expression of IFN-γ, CXCL9 and CXCL10 mRNA (*p* ≤ 0.05) (Figure 7C–E). For CXCL9 and CXCL10, LF-Ag/AuNPs showed better upregulation of mRNA expression in comparison to lactoferrin (*p* ≤ 0.05) (Figure 7C,D). For IFN-γ, this effect was observed only for lactoferrin modified silver nanoparticles (*p* ≤ 0.05) (Figure 7E).

## 4. Discussion

This study demonstrates anti-HSV-2 activity of lactoferrin functionalized silver and gold nanoparticles in the in vitro infection of different types of human keratinocytes but also in the treatment of in vivo HSV-2 infection in mice. The in vitro and in vivo models of HSV-2 infection were chosen on the basis of the results previously obtained in our group [14,17,18,19]. We have demonstrated that combination of antiviral compound, tannic acid (TA), with silver nanoparticles boosts antiviral properties several times [14,17,18]. This effect was due to a better interaction between tannic-acid modified silver nanoparticles and the virus surface [17,18], in comparison to tannic acid itself. Furthermore, TA-AgNPs worked efficiently when applied both as a colloid or in an appropriate mucoadhesive gel to treat HSV-2 infection in the mouse model [19]. However, the antiviral properties of TA-AgNPs depended strongly on the size of nanoparticles with 20–40 nm silver nanoparticles being the most effective without undesired toxic and proinflammatory properties when applied in vivo [14,20].

Here, we observed the same characteristics of small AgNPs (10 nm) as being more toxic than 30 nm AgNPs and 10 nm AuNPs. Toxicity of AgNPs is related with internalization of NPs, which leads to production of ROS, oxidative stress and genotoxic effects. If produced in sufficiently high amounts, ROS can induce cell death by either apoptosis or necrosis [20,21]. The toxicity of silver nanoparticles is also directly related with the ability of a particular cell type to internalize NPs [21]. As previously shown, HaCaT keratinocytes internalize AgNPs very poorly, thus are less sensitive to AgNPs induced toxicity than VK-2-E6/E7 keratinocytes [20]. Here, the differences observed between HaCaT and VK-2 cells sensitivity to AgNPs were insignificant. On the other hand, small (10 nm) AuNPs functionalized with lactoferrin showed very little toxicity for all tested cell types (Table 1). These results were very advantageous as AuNPs sized < 10 nm are more readily accumulated into cell nuclei and organelles, leading to DNA damage [22]. Our results indicate that functionalization of AuNPs sized 10 nm with lactoferrin does not result in toxicity. 

As lactoferrin inhibits different stages of HSV-2 infection, such as attachment, penetration, and cell-to-cell spread of HSV-2, we decided to first perform classical plaque reduction assay in Vero cells (Figure 2). Functionalized silver nanoparticles appeared the most effective in comparison to 10 nm AuNPs (Figure 2). As this assay was performed first by incubating virus with functionalized nanoparticles in RT, we suggest that the presence of silver metal may provide better local environment for interactions between HSV-2 and lactoferrin present on the surface of a metal nanoparticle. Antiviral effects of several cationic peptides, such as LF is dependent on their structure. LF has a relative high net positive charge and its N-terminal region is extremely basic [23]. Therefore, the binding of silver lactoferrin conjugates to the virus surface may be stronger and less prone to dissociation. More research is necessary to decipher the nature of lactoferrin–nanoparticle interaction. Nanoparticles can bind a variety of proteins, forming so called an interfacial corona that provides biological identity and activity to nanoparticles. Kayak et al. demonstrated that adsorption of bovine lactoferrin (BLf) onto AgNP interface is driven by van der Waals interactions and hydrogen bonds, without any significant effect on the protein conformation and stability [24]. BLf also ameliorated the particle-mediated cytotoxicity [24].

Lactoferrin inhibits HSV infection by interfering with virus binding to the target cells but also disturbs intracellular trafficking of HSV virions [25]. We decided to use two different cells lines in order to compare if conjugates can work better than lactoferrin itself in inhibiting of virus attachment and penetration to human keratinocytes. We chose human HaCaT keratinocytes, commonly used to grow HSV and human vaginal VK-2-E6/E7 keratinocytes representing the target cells for HSV-2. Surprisingly, lactoferrin significantly blocked both attachment and penetration of HSV-2 to vaginal keratinocytes rather than to skin keratinocytes (Figure 3 and Figure 4). All lactoferrin conjugates showed very efficient inhibition of HSV-2 attachment and penetration in vaginal keratinocytes (except for 30 nm AgNPs, showing the same inhibition of penetration as lactoferrin itself) but also very good inhibition properties for HaCaT cells in comparison to lactoferrin (Figure 3 and Figure 4). These results prove that lactoferrin conjugates with noble metal nanoparticles can be better antivirals than peptides, especially for cells insensitive to LF activity. 

To further check if lactoferrin conjugates with noble metal nanoparticles can improve inhibition of cell-to-cell spread by lactoferrin, we incubated the infected keratinocytes with conjugates but no significant inhibition was obtained. This is in contrast with previous results showing that lactoferrin can inhibit post-infection spread by interfering with viral glycoprotein complex (gE–gI) [4] or with intracellular pathways [4,6]. Therefore, we can assume that physical form of the conjugates can impede interaction of lactoferrin with glycoproteins involved in cell-to-cell spread or intracellular pathways. 

However, we observed a very interesting effect of pre-treatment with lactoferrin conjugates, which led to significant decrease of HSV-2 infection in VK-2 cells with all tested conjugates and only for conjugated AgNPs in HaCaT cells. Such effect for lactoferrin has not been shown before. Upon cell treatment, lactoferrin is mainly found on the cell surface of cells expressing heparan. This may result from interactions with a highly specific receptor for LF, characterized on several mammalian cell types and tissues [26], but the surface localization of LF is believed to be less important for its antiviral activity [4]. We can speculate that lactoferrin in conjugates may still bind to LF receptors on the cell surface creating in this manner a kind of barrier preventing from HSV-2 infection. This is an additional, advantageous effect of lactoferrin conjugates, which can further contribute to protection of neighboring cells from infection. 

There are numerous studies showing that lactoferrin protects against several, other than HSV-2, viruses. These studies were performed mostly in vitro but there are a few examples where lactoferrin has demonstrated a potent antiviral effect in vivo. For example, lactoferrin given intraperitoneally protects mice against enterovirus and murine cytomegalovirus [27,28]. When deposited in the conjunctival sac it could protect mice against ocular HSV-1 infection [29]. On the other hand, lactoferricin but not lactoferrin was a potent inhibitor of HSV-2 infection in a mouse mode of genital herpes [30]. 

Here, we found that treatment with lactoferrin in 24, 48, and 72h post infection resulted in significant reduction of virus titers both at 3 and 7 day post infection in the vaginal lavages and tissue, respectively (Figure 5). Lactoferrin-functionalized nanoparticles reduced viral titers better than lactoferrin, also in the spinal cords (Figure 5), which makes them promising microbicides. Previously, we demonstrated that tannic acid-modified AgNPs stimulate migration of dendritic cells (DCs) into the vaginal tissue not only early during primary infection but also later, upon re-challenge with the virus [17]. This helped to present the virus antigens both to naïve cytotoxic T cells in the lymph nodes and to memory T cells resulting in the activation of the specific antiviral response [18]. In this study, we also observed significant migration of DCs to both infected and uninfected vaginal tissues stimulated with lactoferrin-modified NPs. It is possible that functionalized nanoparticles can retain lactoferrin present in the vaginal tissues for longer times, thus helping to stimulate both innate antiviral response to HSV-2 (NK cells) and virus-specific CD8+ T cell response (Figure 6). Furthermore, while lactoferrin and lactoferrin-modified nanoparticles differed in the amount of activated innate antiviral response, only nanoparticle conjugates with lactoferrin were able to significantly activate cytotoxic CD8+/granzyme B+ T cells. As shown previously, the aggregates of modified NPs with viral antigens may be better internalized and processed by antigen-presenting cells (APC), thus leading to better activation of immune competent cells downstream. The role of silver and gold nanoparticles as adjuvants for infectious agents is attracting more and attention as well as possible practical uses in vaccines [31,32].

Lactoferrin is able to induce chemokine responses in the genital tract, such as CCL5, one of the major T cell- and NK cell-attracting chemokines [30]. Here, we found that while lactoferrin induced upregulation of CXCL9, CXCL10, and IFN-γ in the vaginal tissues, lactoferrin functionalized nanoparticles induced several times more expression of these chemoattractants for NK cells as well as CD8+ T cells [33], further reflected by increased NK and T cell numbers in the vaginal tissues (Figure 6). Taking into account a well-known fact that small AgNPs can induce cellular toxicity, we also checked for production of inflammatory cytokines, such as IL-1β and TNF-α. The vaginal tissued treated with lactoferrin functionalized 10 nm AgNPs showed significant increase of these two cytokines (Figure 7). TNF-α and IL-1 β are cytokines important for mounting a proper adaptive antiviral response to HSV-2 [34,35]. TNF-α can also induce CCL2 expression and mobilize monocytes/macrophages and T cells through the chemokine receptor CCR2 [35]. However, it is also important to remember that excessive production of proinflammatory cytokines can amplify inflammation triggered by the virally infected cells and result in deregulation of the antiviral response [36]. Therefore, certain caution should be taken when planning in vivo treatment with AgNPs sized < 10 nm. 

## 5. Conclusions

In conclusion, we have demonstrated that lactoferrin functionalized gold and silver nanoparticles have the ability to prevent HSV-2 infection by direct inhibition of virus attachment, penetration and blocking of infection when used in pretreatment. Lactoferrin used in the same concentrations shows significantly inferior antiviral effects, particularly when used in vitro. Therefore, lactoferrin functionalized gold and silver nanoparticles can consist good candidates for effective anti-HSV-2 microbicide to be used in vivo due to their effectiveness at lower concentrations and induction of an anti-viral response.

As both HSV-1 and HSV-2 possess similar structure of surface proteins, lactoferrin functionalized gold and silver nanoparticles can be used not only as a microbicide to treat HSV-2 infections of the anogenital area, but also to treat oral herpes infections in the form of a protective gel or cream to be applied topically. However, further studies are necessary to understand mechanism of antiviral actions of the lactoferrin conjugates.

## Figures and Tables

**Table 1:** Cytotoxicity of lactoferrin-modified 10 nm AuNPs, 10 nm AgNPs, and 30 nm AgNPs or corresponding lactoferrin solution in Vero, VK-2, and HaCaT cells ^1^.

Ag/AuNPs	Vero Cells CC_50_^2^ µg/mL ± SEM	Vero Cells MNTC ^3^ µg/mL ± SEM	HaCaT Cells CC_50_ µg/mL ± SEM	HaCaT Cells MNTC µg/mL ± SEM	VK-2/E6/E7 Cells CC_50_ µg/mL ± SEM	VK-2/E6/E7 Cells MNTC µg/mL ± SEM
LF-AuNPS, 10 nm	39.2 ± 4.7	8.02 ± 1	37.6 ± 4.1	7.78 ± 0.88	28.2 ± 4.2	6.99 ± 1.1
LF-AgNPs, 10 nm	15.01 ± 5.2	6.56 ± 0.45	16.1 ± 2.9	7.2 ± 0.6	11.9 ± 1.4	5.1 ± 0.5
LF-AgNPs, 30 nm	33.3 ± 6.1	9.1 ± 0.78	33.2 ± 7.01	8.1 ± 0.94	27.2 ± 4.3	8.99 ± 0.55
AuNPS, 10 nm	36.8 ± 7.1	9.8 ± 2.5	35.8 ± 7.9	7.21 ± 0.67	26 ± 9.1	7.22 ± 1.2
AgNPs, 10 nm	11.22 ± 3.4	10 ± 3	13.8 ± 1	8.1 ± 0.77	12.3 ± 0.9	7.77 ± 1.4
AgNPs, 30 nm	31.1 ± 2.1	9.39 ± 2.9	30.8 ± 2.2	8.88 ± 0.81	24.2 ± 1.4	8.5 ± 1.9
LF solution ^4^	34.7 ± 7.2	11.45 ± 3.01	39.1 ± 5.4	12.34 ± 2.1	38.5 ± 2.1	16.2 ± 0.9

^1^ The values shown are means from three independent experiments with each treatment performed in triplicate ± SEM. ^2^ Cytotoxic effects were evaluated by MTT assay to determine the concentration of 50% cellular cytotoxicity (CC_50_) of the tested compound and calculated by regression analysis, plotting cytotoxicity percentage to NPs concentration. ^3^ MNTC (maximum non-toxic concentrations) was calculated as NPs concentration with ≤ 20% of non-viable cells. ^4^ Lactoferrin solution in concentration corresponding to concentration present in colloids.

## Data Availability

Data available from authors at request.
